# Supplementary material for: Modeling glioblastoma heterogeneity as a dynamic network of cell states
Source: Mol Syst Biol. 2021 Sep 16;17(9):e10105. doi: 10.15252/msb.202010105 (PMC8444284; doi:10.15252/msb.202010105)
Supplement: Supplementary file 6 — Source Data for Figure 5 [file MSB-17-e10105-s004.zip › Figure5A_sourcedata/GSEA_3071/nosigdb_stateII.GseaPreranked.1622795151171/FELDMAN_2020_G1.html]

Details for gene set FELDMAN\_2020\_G1[GSEA]

|  || Dataset | state23071 |
| Phenotype | NoPhenotypeAvailable |
| Upregulated in class | na\_pos |
| GeneSet | FELDMAN\_2020\_G1 |
| Enrichment Score (ES) | 0.6749117 |
| Normalized Enrichment Score (NES) | 4.226622 |
| Nominal p-value | 0.0 |
| FDR q-value | 0.0 |
| FWER p-Value | 0.0 |
Table: GSEA Results Summary

  

Fig 1: Enrichment plot: FELDMAN\_2020\_G1      
 Profile of the Running ES Score & Positions of GeneSet Members on the Rank Ordered List

  

| PROBE | GENE SYMBOL | GENE\_TITLE | RANK IN GENE LIST | RANK METRIC SCORE | RUNNING ES | CORE ENRICHMENT || 1 | CLSPN |  |  | 1 | 1.984 | 0.0168 | Yes |
| 2 | HIST1H4C |  |  | 2 | 1.902 | 0.0331 | Yes |
| 3 | RRM2 |  |  | 3 | 1.857 | 0.0490 | Yes |
| 4 | ANLN |  |  | 4 | 1.760 | 0.0641 | Yes |
| 5 | HIST1H1B |  |  | 7 | 1.669 | 0.0779 | Yes |
| 6 | TYMS |  |  | 8 | 1.665 | 0.0921 | Yes |
| 7 | CDK1 |  |  | 14 | 1.540 | 0.1041 | Yes |
| 8 | ZWINT |  |  | 16 | 1.510 | 0.1168 | Yes |
| 9 | FEN1 |  |  | 19 | 1.464 | 0.1288 | Yes |
| 10 | UBE2C |  |  | 23 | 1.438 | 0.1404 | Yes |
| 11 | CENPU |  |  | 24 | 1.416 | 0.1525 | Yes |
| 12 | CENPK |  |  | 26 | 1.382 | 0.1641 | Yes |
| 13 | FBXO5 |  |  | 30 | 1.359 | 0.1750 | Yes |
| 14 | USP1 |  |  | 31 | 1.357 | 0.1866 | Yes |
| 15 | RRM1 |  |  | 37 | 1.310 | 0.1966 | Yes |
| 16 | ORC6 |  |  | 39 | 1.308 | 0.2076 | Yes |
| 17 | GINS2 |  |  | 40 | 1.306 | 0.2188 | Yes |
| 18 | TK1 |  |  | 42 | 1.281 | 0.2295 | Yes |
| 19 | AURKB |  |  | 44 | 1.276 | 0.2402 | Yes |
| 20 | MCM7 |  |  | 45 | 1.273 | 0.2511 | Yes |
| 21 | CCNA2 |  |  | 53 | 1.230 | 0.2599 | Yes |
| 22 | DNAJC9 |  |  | 56 | 1.217 | 0.2698 | Yes |
| 23 | PCNA |  |  | 58 | 1.214 | 0.2800 | Yes |
| 24 | HIST1H1C |  |  | 60 | 1.206 | 0.2900 | Yes |
| 25 | MAD2L1 |  |  | 61 | 1.199 | 0.3003 | Yes |
| 26 | UBE2T |  |  | 62 | 1.192 | 0.3105 | Yes |
| 27 | ATAD2 |  |  | 63 | 1.189 | 0.3207 | Yes |
| 28 | SPC25 |  |  | 68 | 1.156 | 0.3296 | Yes |
| 29 | TOP2A |  |  | 72 | 1.132 | 0.3386 | Yes |
| 30 | GMNN |  |  | 83 | 1.108 | 0.3456 | Yes |
| 31 | CEP55 |  |  | 87 | 1.096 | 0.3542 | Yes |
| 32 | HIST1H1E |  |  | 90 | 1.088 | 0.3630 | Yes |
| 33 | VRK1 |  |  | 93 | 1.074 | 0.3717 | Yes |
| 34 | NDC80 |  |  | 94 | 1.074 | 0.3809 | Yes |
| 35 | TMPO |  |  | 99 | 1.056 | 0.3890 | Yes |
| 36 | NCAPG |  |  | 100 | 1.056 | 0.3980 | Yes |
| 37 | DHFR |  |  | 101 | 1.051 | 0.4070 | Yes |
| 38 | MKI67 |  |  | 107 | 1.031 | 0.4146 | Yes |
| 39 | TPX2 |  |  | 111 | 1.025 | 0.4226 | Yes |
| 40 | DUT |  |  | 118 | 1.009 | 0.4298 | Yes |
| 41 | SMC2 |  |  | 123 | 1.005 | 0.4374 | Yes |
| 42 | C12orf75 |  |  | 124 | 1.004 | 0.4460 | Yes |
| 43 | NUF2 |  |  | 125 | 1.004 | 0.4546 | Yes |
| 44 | DEPDC1 |  |  | 126 | 0.999 | 0.4632 | Yes |
| 45 | CKS1B |  |  | 128 | 0.999 | 0.4715 | Yes |
| 46 | KIF20B |  |  | 133 | 0.989 | 0.4790 | Yes |
| 47 | ESCO2 |  |  | 144 | 0.971 | 0.4848 | Yes |
| 48 | CKAP2L |  |  | 148 | 0.959 | 0.4922 | Yes |
| 49 | RANBP1 |  |  | 149 | 0.958 | 0.5004 | Yes |
| 50 | KIF23 |  |  | 161 | 0.936 | 0.5057 | Yes |
| 51 | GTSE1 |  |  | 167 | 0.929 | 0.5124 | Yes |
| 52 | CDCA2 |  |  | 171 | 0.922 | 0.5195 | Yes |
| 53 | CENPM |  |  | 178 | 0.901 | 0.5258 | Yes |
| 54 | H2AFZ |  |  | 213 | 0.864 | 0.5246 | Yes |
| 55 | PBK |  |  | 214 | 0.864 | 0.5320 | Yes |
| 56 | CDCA3 |  |  | 219 | 0.858 | 0.5384 | Yes |
| 57 | CENPH |  |  | 221 | 0.855 | 0.5454 | Yes |
| 58 | ASPM |  |  | 231 | 0.842 | 0.5504 | Yes |
| 59 | MIS18A |  |  | 239 | 0.833 | 0.5558 | Yes |
| 60 | DBF4 |  |  | 240 | 0.832 | 0.5629 | Yes |
| 61 | PRC1 |  |  | 242 | 0.830 | 0.5698 | Yes |
| 62 | LMNB1 |  |  | 251 | 0.824 | 0.5748 | Yes |
| 63 | DNMT1 |  |  | 265 | 0.792 | 0.5783 | Yes |
| 64 | CYR61 |  |  | 267 | 0.791 | 0.5849 | Yes |
| 65 | TUBB4B |  |  | 271 | 0.784 | 0.5908 | Yes |
| 66 | HMGB2 |  |  | 276 | 0.780 | 0.5965 | Yes |
| 67 | SMC4 |  |  | 279 | 0.777 | 0.6027 | Yes |
| 68 | CENPE |  |  | 284 | 0.775 | 0.6083 | Yes |
| 69 | DEK |  |  | 302 | 0.754 | 0.6105 | Yes |
| 70 | CENPA |  |  | 303 | 0.751 | 0.6169 | Yes |
| 71 | KIF4A |  |  | 309 | 0.746 | 0.6220 | Yes |
| 72 | CKS2 |  |  | 312 | 0.745 | 0.6279 | Yes |
| 73 | CENPN |  |  | 320 | 0.737 | 0.6325 | Yes |
| 74 | ANP32B |  |  | 325 | 0.734 | 0.6378 | Yes |
| 75 | CENPW |  |  | 330 | 0.727 | 0.6430 | Yes |
| 76 | AURKA |  |  | 360 | 0.705 | 0.6417 | Yes |
| 77 | DTYMK |  |  | 364 | 0.701 | 0.6470 | Yes |
| 78 | TMEM106C |  |  | 377 | 0.694 | 0.6499 | Yes |
| 79 | SIVA1 |  |  | 380 | 0.690 | 0.6553 | Yes |
| 80 | CCDC34 |  |  | 411 | 0.672 | 0.6535 | Yes |
| 81 | KPNA2 |  |  | 420 | 0.669 | 0.6573 | Yes |
| 82 | RAN |  |  | 424 | 0.667 | 0.6622 | Yes |
| 83 | BUB3 |  |  | 441 | 0.657 | 0.6638 | Yes |
| 84 | SNRPB |  |  | 467 | 0.642 | 0.6630 | Yes |
| 85 | BIRC5 |  |  | 505 | 0.628 | 0.6591 | Yes |
| 86 | SNRPD1 |  |  | 523 | 0.615 | 0.6601 | Yes |
| 87 | RPA3 |  |  | 532 | 0.611 | 0.6633 | Yes |
| 88 | DDX39A |  |  | 542 | 0.605 | 0.6662 | Yes |
| 89 | CENPF |  |  | 575 | 0.588 | 0.6632 | Yes |
| 90 | CCNB1 |  |  | 579 | 0.587 | 0.6675 | Yes |
| 91 | PDLIM7 |  |  | 580 | 0.587 | 0.6725 | Yes |
| 92 | EXOSC8 |  |  | 604 | 0.576 | 0.6717 | Yes |
| 93 | CKAP2 |  |  | 619 | 0.569 | 0.6730 | Yes |
| 94 | HAT1 |  |  | 632 | 0.565 | 0.6748 | Yes |
| 95 | NUSAP1 |  |  | 654 | 0.550 | 0.6743 | Yes |
| 96 | H2AFX |  |  | 671 | 0.544 | 0.6749 | Yes |
| 97 | DLGAP5 |  |  | 712 | 0.529 | 0.6694 | No |
| 98 | PHF19 |  |  | 745 | 0.518 | 0.6658 | No |
| 99 | AHNAK2 |  |  | 758 | 0.514 | 0.6671 | No |
| 100 | PTGES3 |  |  | 763 | 0.512 | 0.6705 | No |
| 101 | CMC2 |  |  | 785 | 0.503 | 0.6695 | No |
| 102 | CDC20 |  |  | 844 | 0.486 | 0.6591 | No |
| 103 | TUBA1B |  |  | 862 | 0.481 | 0.6589 | No |
| 104 | KIF22 |  |  | 975 | 0.450 | 0.6346 | No |
| 105 | HSP90AA1 |  |  | 1008 | 0.443 | 0.6303 | No |
| 106 | PLK1 |  |  | 1009 | 0.442 | 0.6341 | No |
| 107 | SRSF7 |  |  | 1071 | 0.424 | 0.6224 | No |
| 108 | CYCS |  |  | 1113 | 0.415 | 0.6156 | No |
| 109 | MZT1 |  |  | 1219 | 0.393 | 0.5926 | No |
| 110 | PTMA |  |  | 1233 | 0.390 | 0.5926 | No |
| 111 | STMN1 |  |  | 1249 | 0.387 | 0.5922 | No |
| 112 | ANP32E |  |  | 1334 | 0.371 | 0.5742 | No |
| 113 | RNASEH2A |  |  | 1341 | 0.369 | 0.5758 | No |
| 114 | KNSTRN |  |  | 1458 | 0.346 | 0.5496 | No |
| 115 | CALM2 |  |  | 1625 | 0.320 | 0.5105 | No |
| 116 | HMGB1 |  |  | 1720 | 0.308 | 0.4895 | No |
| 117 | CTGF |  |  | 1782 | 0.301 | 0.4767 | No |
| 118 | ILF2 |  |  | 1833 | 0.294 | 0.4667 | No |
| 119 | CMTM7 |  |  | 1845 | 0.293 | 0.4664 | No |
| 120 | IGFBP5 |  |  | 2820 | -0.339 | 0.2240 | No |
| 121 | MIAT |  |  | 2996 | -0.371 | 0.1831 | No |
| 122 | ARL6IP1 |  |  | 3574 | -0.586 | 0.0429 | No |
| 123 | PRCP |  |  | 3775 | -0.766 | -0.0009 | No |
| 124 | SCG2 |  |  | 3975 | -1.137 | -0.0413 | No |
| 125 | IGFBP3 |  |  | 4058 | -1.726 | -0.0472 | No |
| 126 | SCRG1 |  |  | 4070 | -2.021 | -0.0326 | No |
| 127 | PTN |  |  | 4077 | -2.187 | -0.0154 | No |
| 128 | S100B |  |  | 4081 | -2.383 | 0.0043 | No |
Table: GSEA details [plain text format]

  

Fig 2: FELDMAN\_2020\_G1: Random ES distribution      
 Gene set null distribution of ES for **FELDMAN\_2020\_G1**
